# Supplementary material for: A Face-To-Face Comparison of Tumor Chicken Chorioallantoic Membrane (TCAM) In Ovo with Murine Models for Early Evaluation of Cancer Therapy and Early Drug Toxicity
Source: Cancers (Basel). 2022 Jul 21;14(14):3548. doi: 10.3390/cancers14143548 (PMC9325108; doi:10.3390/cancers14143548)

Figure S1. Representative pictures of in ovo tumors for the different cell lines used.

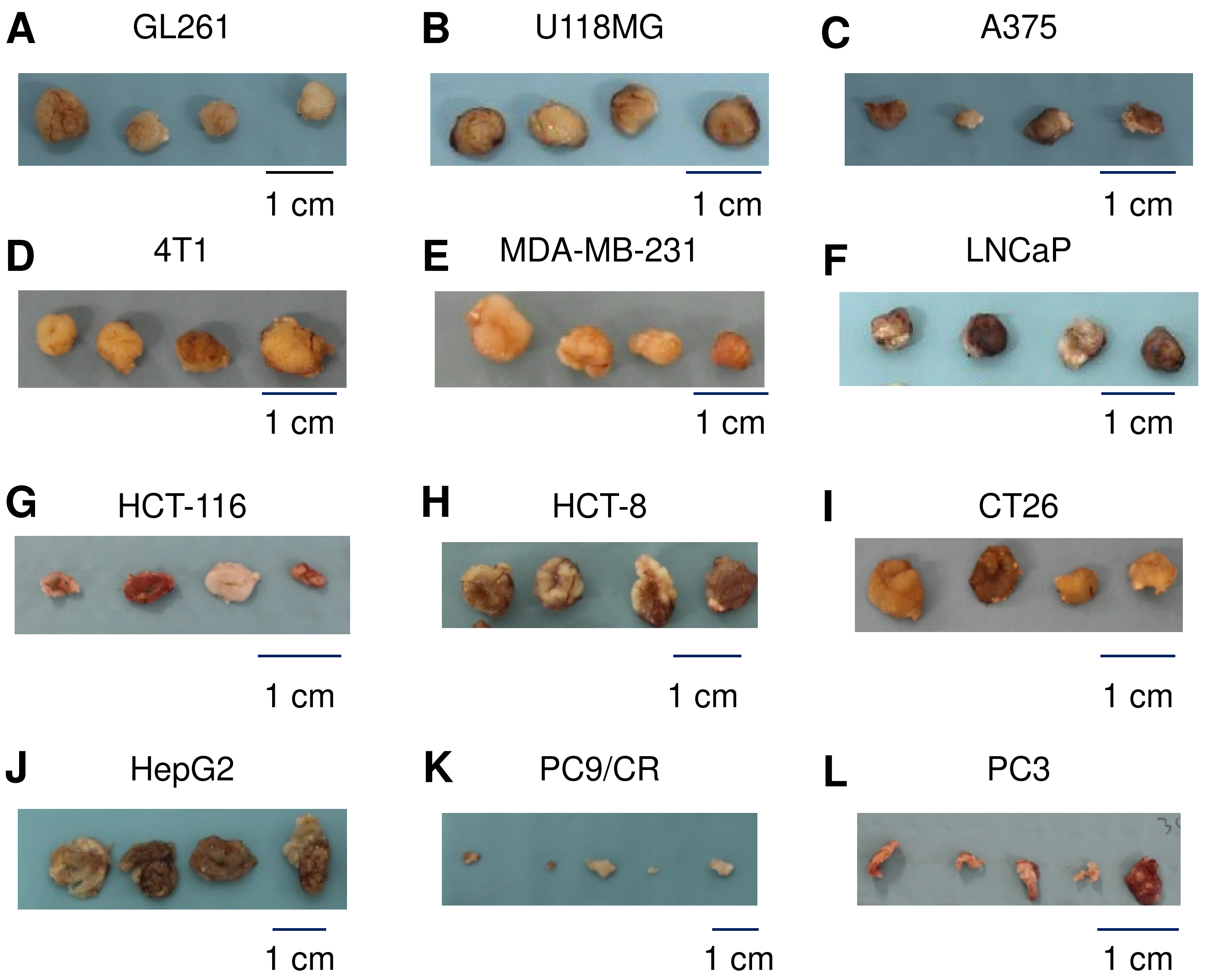

Figure S2. Effect on tumor weight of anticancer drugs in various tumor CAM models.

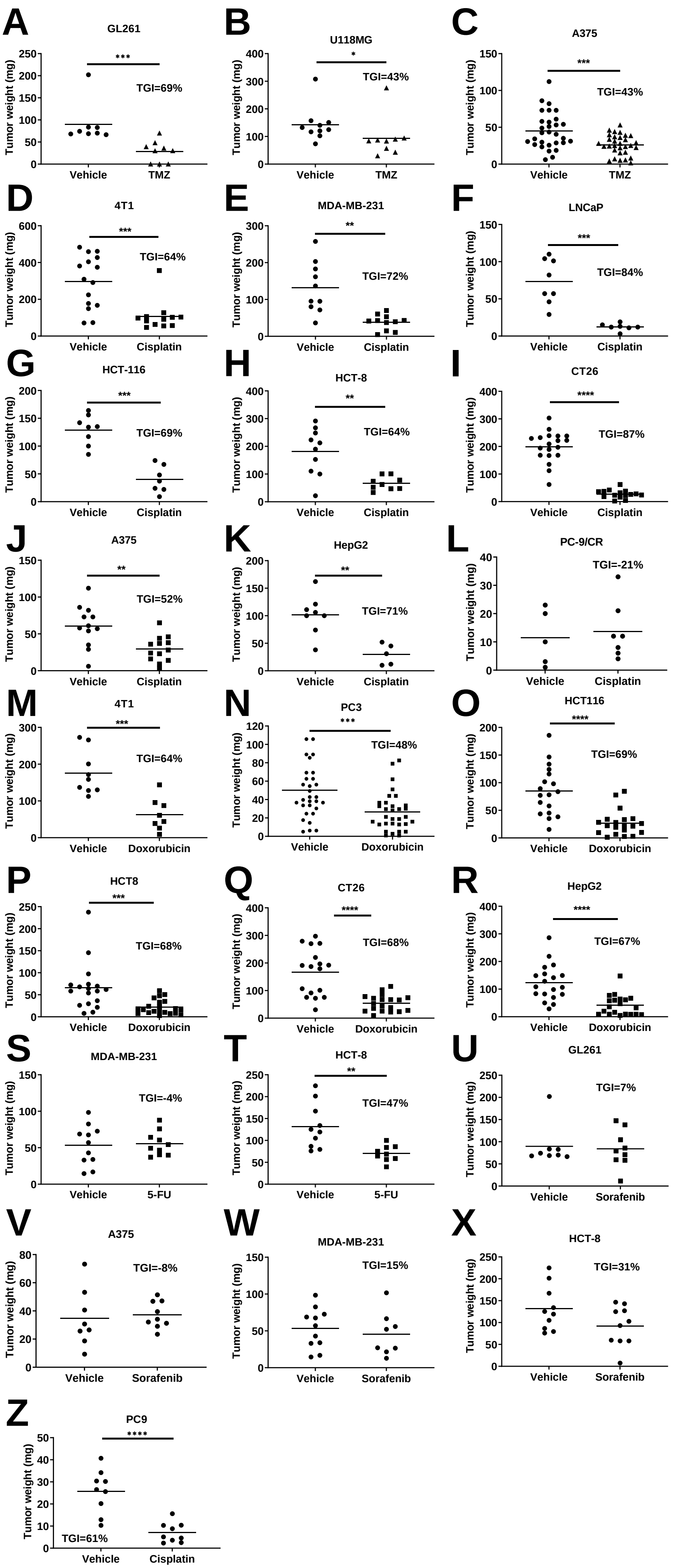

Supplement: Supplementary file 1 [file cancers-14-03548-s001.zip › cancers-1797874-supplementary.pdf]
